# Efficient ^18^F-Labeling of Synthetic Exendin-4 Analogues for Imaging Beta Cells

**DOI:** 10.1002/open.201200014

**Published:** 2012-07-31

**Authors:** Edmund J Keliher, Thomas Reiner, Greg M Thurber, Rabi Upadhyay, Ralph Weissleder

**Affiliations:** a Center for Systems Biology, Massachusetts General Hospital185 Cambridge St, CPZN 5206, Boston, MA 02114 (USA), Fax: (+1) 617-726-8226 E-mail: rweissleder@mgh.harvard.edu; b Department of Systems Biology, Harvard Medical School200 Longwood Ave, Boston, MA 02115 (USA)

**Keywords:** ^18^F-*trans*-cyclooctene, cancer, diabetes, exendin-4, insulinoma, positron emission tomography

## Abstract

A number of exendin derivatives have been developed to target glucagon-like peptide 1 (GLP-1) receptors on beta cells in vivo. Modifications of exendin analogues have been shown to have significant effects on pharmacokinetics and, as such, have been used to develop a variety of therapeutic compounds. Here, we show that an exendin-4, modified at position 12 with a cysteine conjugated to a tetrazine, can be labeled with ^18^F-*trans*-cyclooctene and converted into a PET imaging agent at high yields and with good selectivity. The agent accumulates in beta cells in vivo and has sufficiently high accumulation in mouse models of insulinomas to enable in vivo imaging.

## Introduction

The ability to visualize beta cells noninvasively could have far reaching implications for both biomedical research and clinical practice. Progressive loss of functional beta cell mass (BCM) is the underlying cause of autoimmune type 1 diabetes mellitus, and is also responsible for the secondary failure of clinical drugs in type 2 diabetes. It is widely believed that noninvasive imaging of beta cells could ultimately facilitate not only our understanding of the natural history of islet formation but also the pathophysiology of diabetes. In turn, we would have the capability to diagnose diabetes earlier, monitor the efficacy of widely used drugs, as well as advance the discovery of new therapies. Furthermore, beta cell-specific imaging approaches could be used to diagnose and localize insulinomas and aid the assessment of transplanted islets or pancreata.

In a previous report, we described the development and validation of near infrared fluorescent exendin-4 analogues for imaging beta cells at single cell resolutions,^1^ and for fiber-optic, endoscopic or intraoperative imaging.^2^ We showed that one lead agent, derived from exendin-4 (E4_K12_-FL), had sub-nanomolar EC_50_ binding concentrations and high specificity. In addition, its binding could be inhibited by glucagon-like peptide 1 (GLP-1) receptor agonists. Following intravenous administration to mice, pancreatic islets could be readily distinguished from exocrine pancreas, achieving target-to-background ratios of 6:1. Serial imaging subsequently revealed rapid accumulation kinetics (with initial signal in the islets detectable within 3 min and peak fluorescence occurring within 20 min of injection). Such properties make this an ideal agent for in vivo imaging. Together with other reports of various exendins labeled with chelates,^3^–^9^ we hypothesized that ^18^F-labeled exendin-4 analogues could be used for noninvasive imaging with positron emission tomography-computed tomography (PET–CT). While two approaches of ^18^F-labeling have been recently reported,^10^, ^11^ the ^18^F-conjugation methods used in these studies do not appear to have been used in concert with removal of unreacted material via bioorthogonal scavenging resins.^12^ In this study, we started with a cysteine (C12) version of our previously validated exendin-4 (E4_K12_), by exchanging the lysine at position 12 with a cysteine. Using bioorthogonal labeling strategies employing ^18^F-*trans*-cyclooctene (^18^F-TCO) and tetrazine (Tz) modified molecules,^13^–^15^ we report the facile synthesis and purification of ^18^F-labeled exendin-4. The described reaction demonstrated fast reaction times (20 min), high purity as well as specific activity. Given that the ultimate goal is to translate this technology to the clinic, a lead ^18^F-labeled compound was subsequently applied to PET–CT imaging of insulinoma in a mouse model. Pharmacokinetic modeling, the plasma clearance and tracer-uptake data obtained from these experiments were subsequently used for extrapolation to humans.

## Results and Discussion

**Scheme 1:**
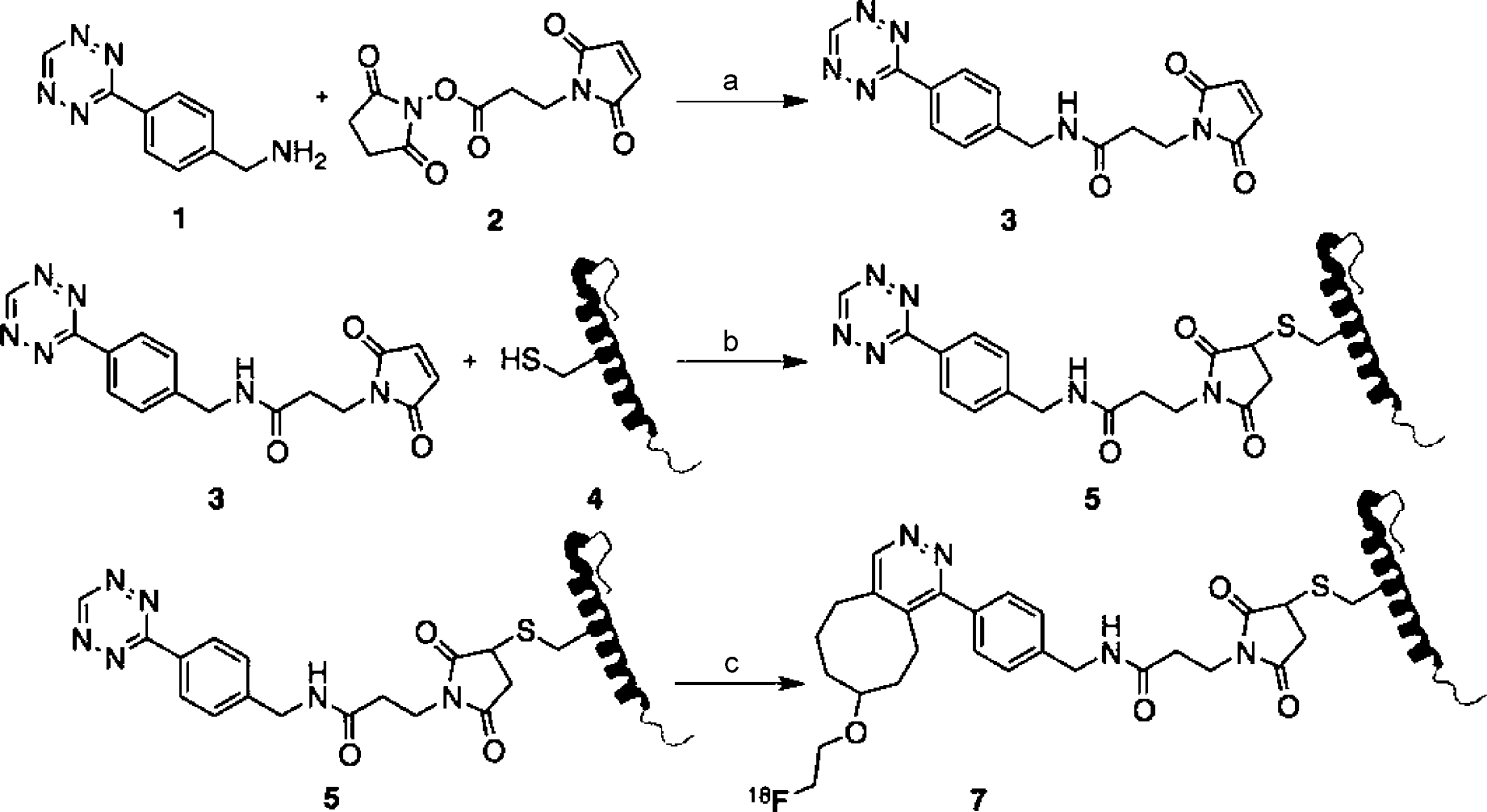
Synthetic scheme for the synthesis of radiolabeled ^18^F-E4_Tz12_ **7**. *Reagents and conditions*: a) triethylamine, acetonitrile/dimethylformamide (4:1), 1 h, 68 %; b) 1×PBS/dimethylformamide (20:1), 3 h, 29 %; c) ^18^F-TCO, 1×PBS/DMSO (1:4), 20 min, 45 % dcRCY.

**Figure 1:**
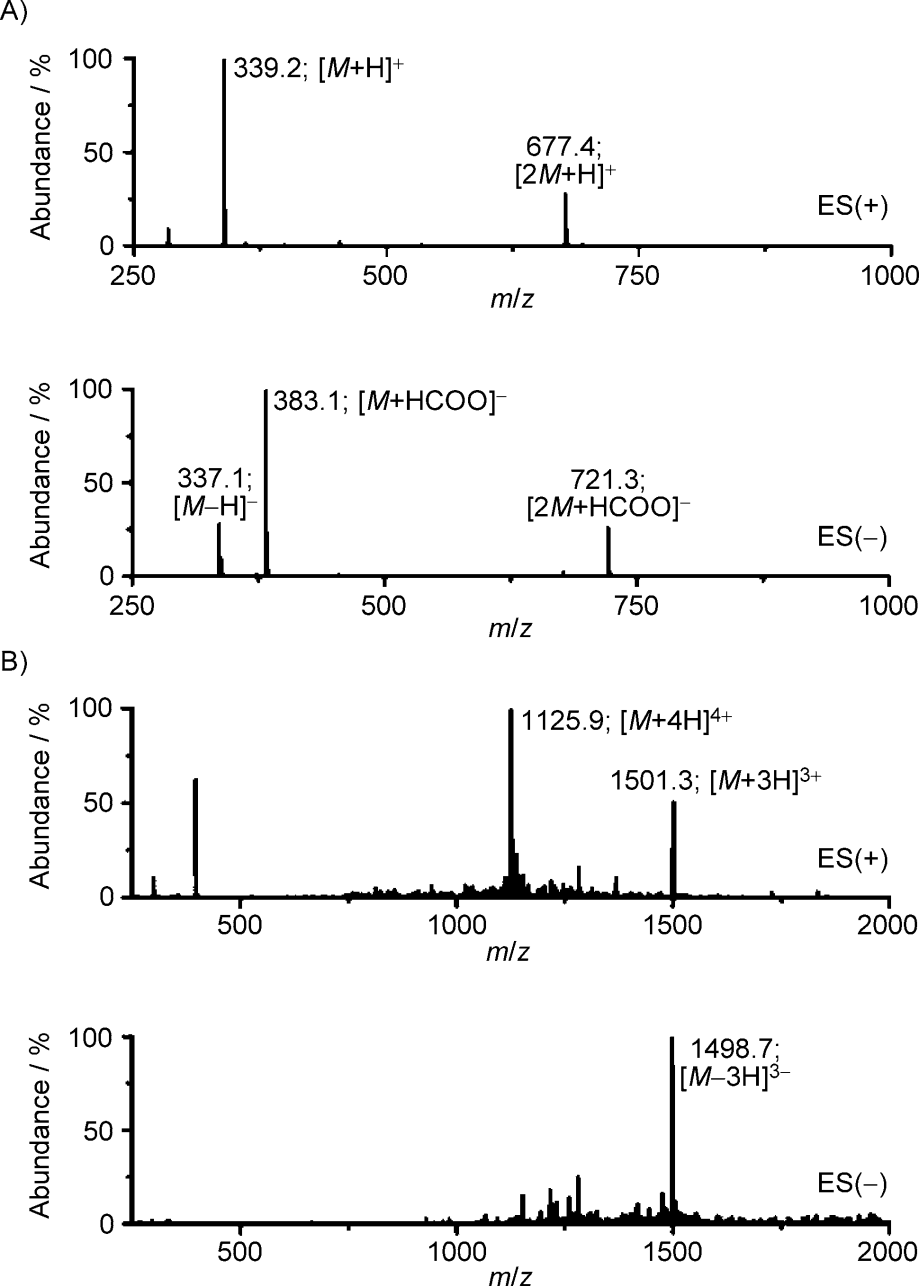
Mass spectra analysis. LC–ESI-MS traces of A) maleimido-Tz **3** and B) E4_Tz12_ **5**.

We previously demonstrated that modification of the exendin-4 amino acid sequence at position 12 does not result in perturbation of the molecule’s intrapancreatic binding, selectivity or specificity for the GLP-1 receptor. In order to translate this finding into a noninvasive ^18^F-PET probe, we designed the cysteine-tetrazine (Tz) cross-linker, maleimide-Tz **3** (Scheme 1). The compound was synthesized from the literature-known Tz amine **1**^16^ and the maleimide-NHS ester **2**^17^ in 68 % isolated yield. This crosslinker readily reacted with E4_C12_, an exendin-4-related peptide in which the natural lysine at position 12 (K12) was exchanged for a cysteine (C12) yielding the bioorthogonally reactive Tz-labeled peptide E4_Tz12_ **5**. Figure 1 shows liquid chromatography–mass spectrometry (LC–MS) traces of both maleimide-Tz **3** (Figure 1 A) and E4_Tz12_ **5** (Figure 1 B), which confirm the identities of the cold precursors.

Similar to the techniques used for small-molecule radiolabeling, we subsequently incubated E4_Tz12_ **5** with ^18^F-*trans*-cyclooctene (^18^F-TCO) **6.**^12^ The radiolabeled bioorthogonally reactive prosthetic group ^18^F-TCO **6** was synthesized in 46 % decay-corrected radiochemical yield (dcRCY) by nucleophilic substitution of the tosylate precursor with ^18^F-fluoride in the presence of tetrabutylammonium bicarbonate (TBAB), as previously described.^13^ ^18^F-TCO **6** (≥94 % pure after HPLC purification) and E4_Tz12_ **5** were then combined in dimethyl sulfoxide (DMSO; 1000 μCi [37 MBq] and 5.5 nmol, respectively) and stirred vigorously for 20 min before yielding a mixture of ^18^F-E4_Tz12_ **7** and unlabeled **5**. Removal of **5** with TCO-modified scavenger resin^12^ followed by centrifugal filtration, provided desired ^18^F-E4_Tz12_ **7**. Simple dilution with phosphate buffered saline (1×PBS) afforded the material ready for injection. The octanol/water and octanol/1×PBS partition coefficients (log*P*) were determined and found to be −1.56±0.06 and −1.75±0.07, respectively, indicating good water solubility.

**Figure 2:**
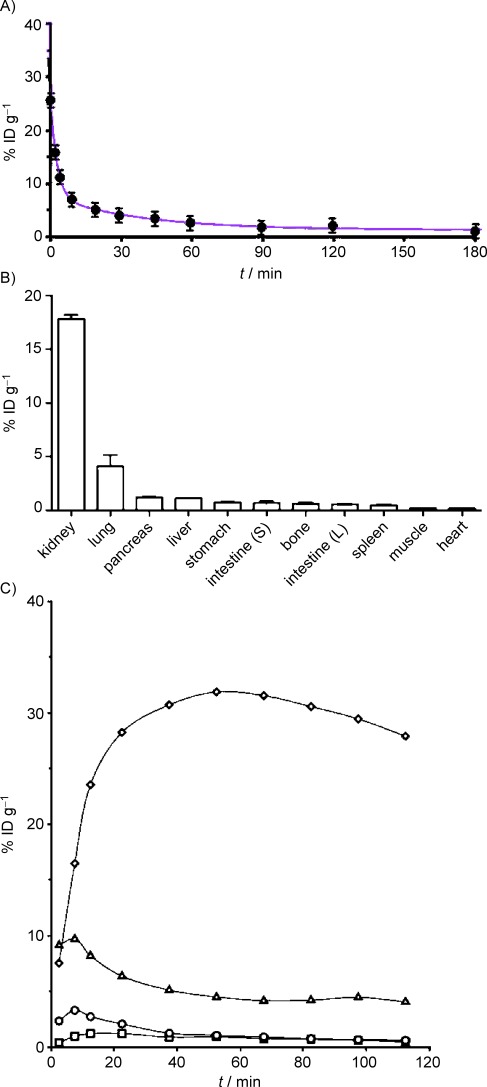
Pharmacokinetics of ^18^F-E4_Tz12_ **7**. A) Blood half-life, *t*_1/2_(weighted)= 6.8±1.1 min and B) biodistribution (at 3 h) of ^18^F-E4_Tz12_ **7** after intravenous administration [injected dose per gram of tissue (% ID g^−1^)]. C) Time–activity curves of ^18^F-E4_Tz12_ **7** obtained from microPET scans of kidney (◊), liver (▵), blood (○), muscle (□).

The blood half-life of ^18^F-E4_Tz12_ **7** was determined through serial retro-orbital bleeds, and the individual data points were then fitted using a biexponential decay curve. This resulted in a weighted half-life (*t*_1/2_) for ^18^F-E4_Tz12_ **7** of 6.8 min [*t*_1/2_(slow)=26.8 min (20 %); *t*_1/2_(fast)=1.9 min (80 %); *R*^2^ of 0.991; Figure 2 A]. Biodistribution of ^18^F-E4_Tz12_ **7** showed dominant renal and hepatobiliary excretion of the compound, with the majority accumulating in the kidneys (17.8±0.6 % injected dose per milligram [% ID g^−1^]), urine, and bowel. Data from dynamic microPET scans generated time–activity curves (Figure 2 C) which support the ex vivo excretion profiles. Tissue levels of the compound were highest in the lungs (4.1±1.5 % ID g^−1^) and pancreas (1.2±0.1 % ID g^−1^); although, uptake of ^18^F-E4_Tz12_ **7** was found to be significantly lower in the pancreata of mice that had been preinjected with cold exenatide (Byetta®, 0.36±0.05 % ID g^−1^). Accumulation in the bone was low (0.6±0.1 % ID g^−1^), indicating minimal defluorination of ^18^F-E4_Tz12_ **7**.

**Figure 3:**
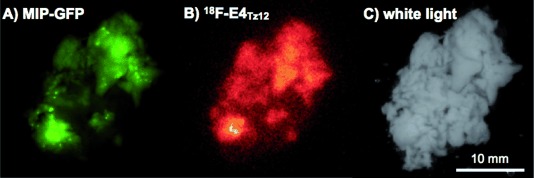
Correlation of MIP-GFP expression and ^18^F-E4_Tz12_ **7** distribution in resected pancreata. A) Green fluorescence indicates islets of Langerhans in a MIP-GFP mouse. B) Distribution of ^18^F-E4_Tz12_ **7** in vivo in a mouse pancreas, as assessed by autoradiography imaging. C) White-light imaging of the resected pancreas.

To determine the intra-pancreatic distribution of the compound (islets of Langerhans comprise only 1–2 % of the pancreatic mass), we performed autoradiography. We injected ^18^F-E4_Tz12_ **7** (92±12 μCi [3.40±0.44 MBq]) via tail vein into transgenic mice that express enhanced green fluorescent protein (eGFP) under the control of the mouse insulin promoter [mouse insulin promoter (MIP)-green fluorescent protein (GFP)].^18^ After 3 h, the mice were euthanized, and their pancreata excised. The pancreata were then imaged using surface reflectance imaging (to show the islet distribution) before being exposed for autoradiography (to show the distribution of ^18^F-E4_Tz12_ **7**). Figure 3 shows good colocalization between the fluorescence of the GFP islet and the autoradiographic signal from ^18^F-E4_Tz12_ **7** with a Pearson’s coefficient of 0.83±0.04 (*R*_coloc._). Based on micro-dissected specimens and target-to-background ratios, we calculated a concentration of approximately 40 % ID g^−1^) in the islets.

**Figure 4:**
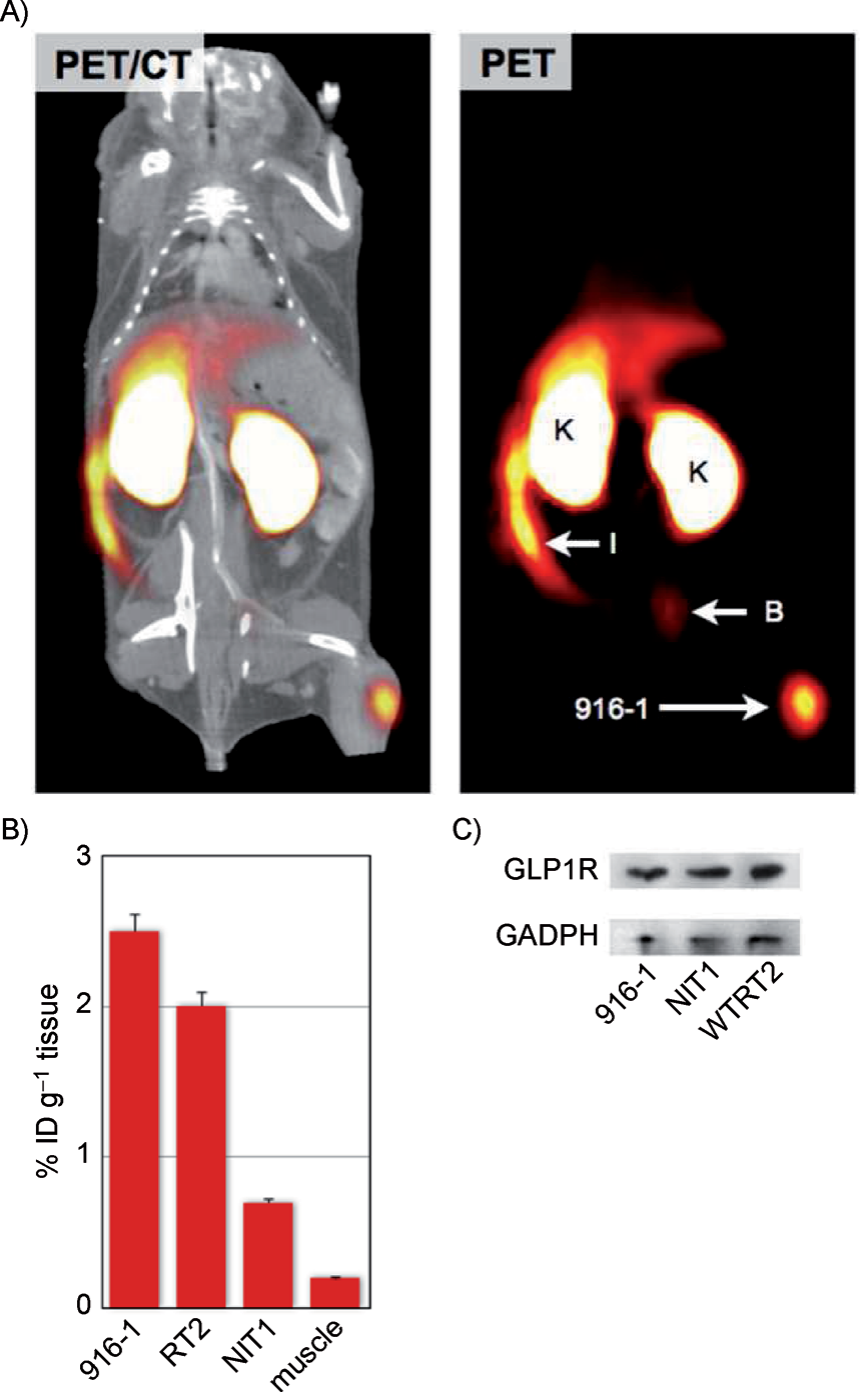
In vivo imaging of insulinoma. A) PET-CT and PET only scans of ^18^F-E4_Tz12_ **7** in a C57BL/6 mouse bearing 916-1 tumor xenografts. B) Average uptake of ^18^F-E4_Tz12_ **7** in different tumors and in muscle tissue [injected dose per gram of tissue (% ID g^−1^)]; tumor xenograft (916-1), intestines (I), kidney (K), bladder (B). C) Western blot of 916-1, NIT1, and WTRT2 cell lines against GLP-1R and GAPDH.

To determine the utility of ^18^F-E4_Tz12_ **7** for insulinoma detection, we tested it in different murine models: NIT-1, 916-1 or WTRT2 mouse insulinoma xenografts. These cell lines were chosen for their elevated GLP1R expression as verified by Western blot (Figure 4 C). For tumors, uptake values of 2.5 % ID g^−1^ (916-1), 2.0 % ID g^−1^ (WTRT2) and 0.7 % ID g^−1^ (NIT-1) were obtained, which allowed them to be detected by whole body PET imaging (Figure 4 A and B). Tumor-to-muscle ratios from ex vivo scintillation counting data were 13.4, 10.5, and 14.6 for 916-1, WTRT2 and NIT-1, respectively. In all cases, preinjection of cold exenatide (250 μL, 60 μm) resulted in a significant reduction of the standard uptake values (916-1: 82 % reduction; WTRT-2: 54 % reduction; NIT-1: 62 % reduction). In contrast, muscle standard uptake values were not affected by preinjection with cold exenatide (0.11 % ID g^−1^) This confirms the applicability and selective uptake of ^18^F-E4_Tz12_ **7** as a targeted probe for GLP-1 receptor-rich tissues.

Ultimately, these agents are being developed for their clinical application. While their clearance is very rapid in mice (80 % with a 1.9 min half-life and 20 % with a 26.8 min half-life for ^18^F-E4_Tz12_ **7**), we were interested in determining the optimal clearance kinetics in humans. A compartmental pharmacokinetic model was thus developed to extrapolate our results from mice. The advantage of this model is that some of the parameters (e.g., plasma clearance) that vary between species can be scaled up, while others (e.g., the binding rate constants and radioactive decay half-life) are kept constant.

Using clinical data available for exenatide, the plasma concentration after continuous infusion^19^ was fit to a two-compartmental model, in order to predict the percent clearance of a bolus imaging dose. The results indicated that 73 % of the imaging agent dose redistributes to peripheral tissues with a rapid 1 min half-life, while the remaining 27 % clears with a 63 min half-life. This is close to the percent clearance observed with inulin in humans following an intravenous bolus injection (76 % with a 10 min half-life and 24 % with a 86 min half-life^20^); the model therefore provides a reasonable estimate of clearance.

**Figure 5:**
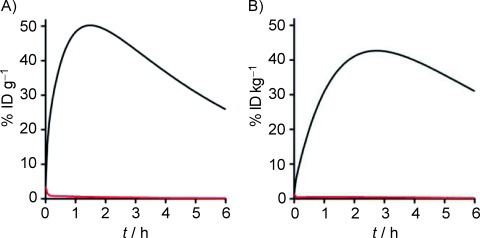
Extrapolation of uptake in human pancreata using pharmacokinetic modeling. A) The efficiency of uptake in the islet (—) versus the exocrine pancreas (—) is shown for the mouse using a compartmental model. Initial rate constants were adjusted to fit experimental data. B) The mechanistic rate constants (from mice) were combined with a fitted clearance in humans (based on clinical data), to estimate uptake and clearance of the compound in islets (—) and exocrine pancreas (—) following injection of a bolus dose [injected dose per gram of tissue (% ID g^−1^)]. Due to differences in body weight, the human data is expressed as injected dose per kilogram of tissue (% ID kg^−1^).

The exchange rate of the compound between the plasma and extracellular space was subsequently estimated from literature values^21^ and adjusted to fit our experimental results in mice (Figure 5 A). The results in Figure 5 B show estimates of human uptake and clearance, based on clinical data, and using mechanistic rate constants from mice. In both cases, the specific uptake of the compound in islets is significantly higher than in the exocrine pancreas due to its specific target binding.

The GLP-1 receptor is highly expressed in beta cells within the islets of Langerhans as well as in functioning beta cell islet tumors (insulinomas). GLP-1 analogues are a new class of peptide-based drugs used for the treatment of diabetes. Exenatide, the first FDA approved GLP-1 analogue, is a synthetic version of exendin-4. It is a 39-amino acid peptide isolated from the saliva of the Gila monster (*Heloderma suspectum*) and contains 53 % sequence homology with GLP-1. A recent crystal structure of the extracellular domain of the GLP-1 receptor showed the binding mode of exendin-4 (amino acids 9–39).^22^ From this crystal structure, it was clear that lysine 12 (K12) is not involved in binding to the GLP-1 receptor domain. Moreover, it explains why K12-modified exendins retain high affinity for the receptor.^1^, ^2^ Our results further demonstrate that K12 modification with tetrazines are not only stable but allow rapid site-specific and high-yield fluorinations. Tetrazine functionalization of the peptide also allows removal of unreacted starting material with the complimentary *trans*-cyclooctene beads, an option not available to other current ^18^F or metal chelation labeling strategies. The resulting compounds exhibit appropriate pharmacokinetics for PET imaging of beta cells in a mouse model.

In an effort to predict the compound’s kinetics in humans, we applied pharmacokinetic modeling and allometric scaling.^23^, ^24^ In the mouse, the synthesized compound had a weighted half-life (*t*_1/2_) of 6.8 min. Using our modeling and scaling approach, we predicted a *t*_1/2_ value of 18 min in human. Importantly, we found that this molecule size has a beta phase clearance half-life of 63 min. Agents with clearance rates that are much slower than the radioactive half-life could have a background that is too high during the imaging window. Conversely, agents that clear much faster than the radioactive half-life could have inefficient accumulation within the target tissue. Given that the decay of ^18^F is 109.8 min, our modeling indicates that this compound would have close to ideal clearance for human imaging. The pharmacokinetic modeling also indicates that further improvements in linker modification could reduce exocrine uptake and improve detection sensitivity. For example, by using bioorthogonal chemistry, which allows facile modulation of the linkers, further improvements in the reaction kinetics, stability and biocompatibility of the compound could be achieved.^25^, ^26^

## Experimental Section

### Chemistry

**General**: Unless otherwise noted, all reagents were purchased from Sigma–Aldrich (St. Louis, MO, USA) and used without further purification. Exendin-4 (exenatide, Byetta®) was obtained from Amylin/Eli Lilly (San Diego, CA, USA). E4_C12_ (4163 g mol^−1^; HGEGTFTSDLSCQMEEEAVRLFIEWLKNGGPSSGAPPPS) was obtained from Genscript (Piscataway, NJ, USA). [^18^F]-Fluoride (n.c.a.) was purchased from PETNET Solutions (Woburn, MA, USA). 3-maleimido-propanoic acid succinimidyl ester **1**, tetrazine (Tz) amine **2** and ^18^F-*trans*-cyclooctene (^18^F-TCO) **4** were synthesized as described elsewhere.^13^, ^16^, ^17^, ^27^ High performance liquid chromatography–electrospray ionization mass spectrometry (HPLC–ESI-MS) analyses and HPLC purifications were performed on a Waters LC-MS system (Milford, MA, USA). For LC–ESI-MS analyses, a Waters XTerra® C18 5 μm column was used. For preparative runs, an Atlantis® Prep T3 OBD™ 5 μm column was used. High-resolution ESI mass spectra were obtained on a Bruker Daltonics APEXIV 4.7 Tesla Fourier Transform ion cyclotron resonance mass spectrometer (FT-ICR-MS) in the Department of Chemistry Instrumentation Facility at the Massachusetts Institute of Technology. Proton nuclear magnetic resonance (^1^H NMR) spectra were recorded on a Varian AS-400 (400 MHz) spectrometer. Chemical shifts for protons are reported in parts per million (ppm) and are referenced against the [D_6_]acetone lock signal (^1^H, 2.05 ppm). NMR data are reported as follows: chemical shift, multiplicity (s=singlet, d=doublet, t=triplet, m=multiplet), coupling constants (Hz) and integration.

**3-maleimido propanamide-tetrazine (maleimido-Tz) 3**: A solution of 3-maleimido-propanoic acid succinimidyl ester **1** (5 mg, 19 μmol, 20 mg mL^−1^, 250 μL in dimethylformamide (DMF) was added to a solution of Tz amine **2** (3.5 mg, 19 μmol) and Et_3_N (5.3 μL) in MeCN (1 mL), and the resulting reaction mixture stirred at RT for 1 h. Volatiles were removed in vacuo and the crude product purified using HPLC to give compound **3** as a pink solid (4.4 mg, 13 μmol, 68 %): ^1^H NMR (400 MHz, [D_6_]acetone): *δ*=10.43 (s, 1 H), 8.52 (d, ^3^*J*_HH_=8.3, 2 H), 7.78 (m, 1 H), 8.58 (d, ^3^*J*_HH_=8.2, 2 H), 6.86 (s, 2 H), 4.52 (d, ^3^*J*_HH_=6.0, 2 H), 3.80 (t, ^3^*J*_HH_=7.4, 2 H), 2.59 ppm (t, ^3^*J*_HH_=7.4, 2 H); LC–ESI-MS(+): *m*/*z* (%): 339.2 (100) [*M*+H]^+^, 677.4 (29) [2*M*+H]^+^; LC–ESI-MS(−): *m*/*z* (%): 337.1 (29) [*M*−H]^−^, 383.1 (100) [*M*+HCOO]^−^, 721.3 (27) [2*M*+HCOO]^−^; HRMS-ESI: *m*/*z* [*M*−H]^+^ calcd for [C_16_H_14_N_6_O_3_Na]^+^ 361.1020, found 361.1013 [*M*+Na]^+^.

**E4_Tz12_** **5**: A solution of maleimido-Tz **3** (50 μL 10 mm) in DMF was added to a solution of E4_C12_ **4** (3.0 mg, 0.7 μmol) in 1×PBS (1000 μL), and the resulting solution was stirred at RT for 3 h. The reaction mixture was purified using an Amicon® Ultra 3 kDa centrifugal filter (Millipore, Carrigtwohill, Ireland) before being subjected to HPLC purification, yielding compound **5** as a rose-colored solid (0.8 mg, 0.2 μmol, 29 %): LC–ESI-MS(+): *m*/*z* (%): 1125.9 (100) [*M*+4H]^4+^, 1501.3 (51) [*M*+3H]^3+^; LC–ESI-MS(−): *m*/*z* (%): 1498.7 (100) [*M*−3H]^3−^.

^**18**^**F-E4_Tz12_** **7**: 2-[^18^F]-(*E*)-5-(2-Fluoroethoxy)cyclooct-1-ene (^18^F-TCO) was prepared in a similar manner to previously described procedures^13^ employing a Synthra RN Plus automated synthesizer (Synthra GmbH, Hamburg, Germany) operated by SynthraView software in an average time of 102 min. The synthesizer reagent vials were filled with the following: A2 with MeCN (350 μL), A3 with (*E*)-2-(cyclooct-4-enyloxy)ethyl 4-methylbenzenesulfonate (2.0 mg, 12.3 μmol) in DMSO (400 μL), A5 with MeCN (150 μL), and B2 with H_2_O (800 μL). The starting activity well was filled with [^18^F]-F^−^ (n.c.a.) (2072 MBq, 56±15 mCi) in H_2_^18^O (500–1000 μL), tetrabutylammonium bicarbonate (TBAB, 250 μL, 75 mm in H_2_O), and MeCN (200 μL). The [^18^F]-F^−^/TBAB solution was transferred to the reaction vessel and dried by azeotropic distillation with MeCN. After drying, TCO-tosylate (2 mg, 15 mm) in DMSO was added and heated to 90 °C for 10 min. After cooling to 30 °C, the mixture was filtered through an Alumina-N cartridge (100 mg, 1 mL, Waters) into reaction vessel 2. The Alumina-N cartridge was washed with MeCN (150 μL) and the combined filtrates were then diluted with H_2_O (800 μL). This solution was subsequently subjected to preparative HPLC purification (MeCN/H_2_O, 50:50). ^18^F-TCO was collected (*t*_R_=13.5 min) in 5–6 mL of solvent and isolated by manual C18 solid phase extraction. It was then eluted with DMSO (2×450 μL) to give 10.1±5.9 mCi of ^18^F-TCO in 46.1±12.2 % (*n*=4) decay-corrected radiochemical yield (dcRCY) in an average time of 102 min (once drying of [^18^F]-F^−^ (n.c.a.) had ended). Analytical HPLC demonstrated >94 % radiochemical purity of ^18^F-TCO.

E4_Tz12_ **5** (5.5 nmol, 1 mm in DMSO) was added to the ^18^F-TCO **6** in DMSO. After stirring at RT for 20 min, TCO-beads (150 uL suspension of 10 mg mL^−1^; TCO loading: 13 nmol mg^−1^) were added to the mixture and stirred for 20 min. The reaction mixture was filtered using an Amicon® Ultra 3 kDa centrifugal filter (Millipore, Carrigtwohill, Ireland) to give ^18^F-E4_Tz12_ **7 (**1.8±0.9 mCi, 46.7±17.3 % (*n*=4) dcRCY).

^18^F-E4_Tz12_ **7** (approx. 14 μCi [0.52 MBq]) in DMSO/1×PBS (4:1, 5 μL) was added to octanol (500 μL) and H_2_O (MilliQ, 500 μL) in a 1.5-mL microcentrifuge tube. The mixture was vortexed for 1 min at RT and centrifuged (15 000 rpm, 5 min). After centrifugation, 100-μL aliquots of both layers were measured using a γ-counter. The experiment was carried out in quintuplicate. This experiment was repeated with octanol/1×PBS (1:1, 1000 μL).

### Biological Evaluation

**Cell lines**: We chose three different insulinoma tumor cell lines (NIT-1, WTRT2, 916-1), to correlate imaging findings and to elucidate how ^18^F-E4_Tz12_ behaves in different insulinoma tumor environments. Both WTRT2 and 916-1 were generously provided by Johanna Joyce (Memorial Sloan–Kettering Cancer Center, New York City, USA). NIT-1 was obtained from the American Type Culture Collection (ATCC, Manassas, VA, USA). WTRT2 and 916-1 were cultured in Dulbecco’s modified Eagle medium (DMEM) supplemented with fetal bovine serum (10 %), L-glutamine, penicillin (100 I.U.), and streptomycin (100 μg mL^−1^). NIT-1 were cultured in F-12K medium (Kaighn’s Modification of Ham’s F-12 Medium, ATCC, Manassas, VA) supplemented with fetal bovine serum (10 %), sodium bicarbonate (2 %), L-glutamine, penicillin (100 I.U.), and streptomycin (100 μg mL^−1^). All cell lines were cultured at 37 °C and 5 % CO_2_.

**Western Blot**: 916-1, WTRT2, and NIT-1 cells seeded into six-well plates were washed twice with ice-cold 1×PBS and lysed on ice for 10 min with ice-cold RIPA lysis buffer (100 μL) supplemented with a 100-fold dilution of protease inhibitor cocktail for mammalian cells (Sigma–Aldrich). The lysate was centrifuged (10 min, 10 000 rcf) and the supernatant collected. Protein concentrations were determined using bicinchoninic acid (BCA) protein assays (Pierce, Rockford, IL, USA). Cell lysates (10 μg) were subjected to SDS-PAGE, followed by immunoblotting using anti-GLP-1R antibody (#39072, Abcam, Cambridge, UK), goat-anti-rabbit secondary (Jackson ImmunoResearch, West Grove, PA, USA), and detection with chemiluminescence (Picowestern ECL substrate, Pierce). Blots were stripped using Restore Stripping Buffer (Thermo Scientific), labeled with anti-GAPDH antibody (AF 5718, R&D Systems) followed by detection with chemiluminescence.

**Mice**: Experiments were performed in Nu/Nu mice (from Massachusetts General Hospital, Boston, MA; for tumor implantations and imaging; *n*=6), C57BL/6 (B6) mice (from The Jackson Laboratory, Bar Harbor, ME; for biodistribution and pharmacokinetics; *n*=8), or B6.Cg-Tg(Ins1-EGFP)1Hara/J mice (from The Jackson Laboratory, Bar Harbor, ME; for autoradiography/surface reflectance imaging; *n*=3).^18^ B6.Cg-Tg(Ins1-EGFP)1Hara/J mice express the enhanced green fluorescent protein (eGFP) in the islets under the control of the mouse insulin 1 promoter (MIP-GFP). For all surgical procedures and imaging experiments, mice were anesthetized with 2.0 % isoflurane in O_2_ at 2.0 L min^−1^. For imaging experiments lasting longer than 1 h, the isoflurane flow rate was reduced to ∼1.0 % isoflurane in O_2_ at 2.0 L min^−1^. Surgeries were conducted under sterile conditions with a zoom stereomicroscope (Olympus SZ61). All procedures and animal protocols were approved by the Massachusetts General Hospital subcommittee on research animal care.

**Whole pancreas islet imaging**: B6.Cg-Tg(Ins1-EGFP)1Hara/J (MIP-GFP) mice^18^ were administered ^18^F-E4_Tz12_ **7** (92±12 μCi [3.40±0.44 MBq]) via intravenous tail-vein injection, and the GPL-1 receptor-specific probe was allowed to accumulate and clear for 3 h. Mice were then euthanized, their organs perfused using 1×PBS (30 mL) and the pancreata harvested. They were subsequently weighed and placed between two glass cover slides using a 1 mm rubber gasket, maintaining a constant thickness. Initially, fluorescence reflectance was recorded by imaging the entire pancreas on an OV110 epifluorescence imager (Olympus America, Center Valley, PA, USA). The pancreata were then transferred to an autoradiography phosphor imaging plate (SI, Molecular Dynamics) and exposed at −20 °C for 12 h before the plate was analyzed using a Typhoon scanner (GE Healthcare). Image analysis was conducted using ImageJA 1.45 software.

^**18**^**F-E4_Tz12_** **7 biodistribution studies**: C57BL/6 (B6) mice were used for blood half-life determinations. Mice were administered ^18^F-E4_Tz12_ **7** (68±12 μCi [2.52±0.44 MBq]) by intravenous tail-vein injection. Blood sampling was performed by retro-orbital puncture using tared, heparinized capillary tubes. Samples were subsequently weighed and activity measured using a Wallac Wizard 3“ 1480 Automatic Gamma Counter (PerkinElmer). Blood half-life data were fitted to a biexponential model using Graphpad Prism 4.0c software (GraphPad Software Inc., San Diego, CA), and results were reported as the weighted average of the distribution and clearance phases. For biodistributions, (B6) mice were intravenously injected via tail vein with ^18^F-E4_Tz12_ **7** (131±18 μCi [4.85±0.67 MBq]). Animals were euthanized at 3 h and their organs perfused using 1×PBS (30 mL). Tissues were subsequently harvested, weighed and their radioactivity counted using a Wallac Wizard 3” 1480 Automatic Gamma Counter. Statistical analysis was performed using Graphpad Prism 4.0c.

**MicroPET-CT imaging**: Mice were imaged by PET-CT using an Inveon small animal microPET scanner (Siemens Medical Solutions). Mice were injected with ^18^F-E4_Tz12_ **7** (557±38 μCi [20.61±1.41 MBq]) via tail-vein injection under isoflurane anesthesia (see above). Acquisition for static microPET images started 2 h post injection and acquisition took approximately 30 min. For dynamic microPET imaging, mice were injected approximately 30 s after the start of microPET acquisition, and data was collected for 2 h. The radioactivity concentration for a tissue was determined by measuring within regions of interest (ROIs) for a given tissue with the units of Bq mL^−1^ min^−1^. A tissue density of 1 g mL^−1^ was assumed and ROIs were converted to Bq g^−1^ min^−1^ and divided by the injected activity to obtain an imaging ROI-derived % ID g^−1^. For GLP-1 receptor blocking experiments, unlabeled exenatide (250 μL, 60 μm) was preinjected 45 min prior to injection of ^18^F-E4_Tz12_ **7**. A high-resolution Fourier rebinning algorithm was used, followed by a filtered back-projection algorithm using a ramp filter, to reconstruct 3D images without attenuation correction. The image voxel size was 0.796×0.861×0.861 mm, for a total of 128×128×159 voxels. Peak sensitivity of the Inveon accounts for 11.1 % of positron emission, with a mean resolution of 1.65 mm. The total counts acquired was 600 million per PET scan. Calibration of the PET signal with a cylindrical phantom containing ^18^F was performed before all scans. CT images were reconstructed using a modified Feldkamp reconstruction algorithm (COBRA) from 360 cone-beam X-ray projections (80 kVp and 500 μA X-ray tube). The isotropic voxel size of the CT images was 60 μm. The reconstruction of data sets, PET-CT fusion, and image analysis were performed using Inveon Research Workplace (IRW) software (Siemens). 3D visualizations were produced using a digital imaging and communications in medicine (DICOM) viewer (OsiriX Foundation, Geneva, Switzerland).

### Modeling

A compartmental model was used to extrapolate results from mouse-imaging studies to humans. The model includes biexponential loss from the plasma compartment (due to redistribution and clearance), and separate compartments for the endocrine and exocrine pancreas. Exchange with the endocrine tissue (islets) was estimated as a function of the vascular surface area-to-volume ratio (measured at 505±146 cm^−1^ using CD31 stained histology slides),^28^ and permeability was estimated at 30 μm s^−1^ (for this sized molecule in the fenestrated capillary bed).^21^ Exocrine pancreas was modeled in a similar manner, while the exchange parameters were adjusted to fit experimental data. Within the compartments, the imaging agent is able to bind the target, dissociate, internalize, and be degraded and washed out.^24^ These rate constants were assumed constant between species. For plasma clearance in humans, the rate constants for exchange and clearance from a two-compartmental model were fit to experimental data taken from patients undergoing an intravenous infusion of exenatide^19^ using a least-squares fitting algorithm in Matlab (Mathworks, Natick, MA, USA). Estimates for humans were obtained by entering the plasma clearance values from human clinical data into the model together with the microscopic transport rates obtained from mouse experiments.

## References

[1] Reiner T, Kohler RH, Liew CW, Hill JA, Gaglia J, Kulkarni RN, Weissleder R (2010). Bioconjugate Chem.

[2] Reiner T, Thurber G, Gaglia J, Vinegoni C, Liew CW, Upadhyay R, Kohler RH, Li L, Kulkarni RN, Benoist C, Mathis D, Weissleder R (2011). Proc. Natl. Acad. Sci. USA.

[3] Brom M, Oyen WJ, Joosten L, Gotthardt M, Boerman OC (2010). Eur J Nucl Med Mol Imaging.

[4] Mukai E, Toyoda K, Kimura H, Kawashima H, Fujimoto H, Ueda M, Temma T, Hirao K, Nagakawa K, Saji H, Inagaki N (2009). Biochem. Biophys. Res. Commun.

[5] Pattou F, Kerr-Conte J, Wild D (2010). N. Engl. J. Med.

[6] Wild D, Wicki A, Mansi R, Behe M, Keil B, Bernhardt P, Christofori G, Ell PJ, Macke HR (2010). J Nucl Med.

[7] Wild D, Mäcke H, Christ E, Gloor B, Reubi JC (2008). N. Engl. J. Med.

[8] Wicki A, Wild D, Storch D, Seemayer C, Gotthardt M, Behe M, Kneifel S, Mihatsch MJ, Reubi JC, Mäcke HR (2007). Clin. Cancer Res.

[9] Wu Z, Todorov I, Li L, Bading JR, Li Z, Nair I, Ishiyama K, Colcher D, Conti PE, Fraser SE, Shively JE, Kandeel F (2011). Bioconjugate Chem.

[10] Kiesewetter DO, Gao H, Ma Y, Niu G, Quan Q, Guo N, Chen X (2012). Eur J Nucl Med Mol Imaging.

[11] Wang Y, Lim K, Normandin M, Zhao X, Cline GW, Ding YS (2012). Nucl Med Biol.

[12] Reiner T, Keliher EJ, Earley S, Marinelli B, Weissleder R (2011). Angew. Chem.

[13] Keliher EJ, Reiner T, Turetsky A, Hilderbrand SA, Weissleder R (2011). ChemMedChem.

[14] Reiner T, Lacy J, Keliher EJ, Yang KS, Ullal A, Kohler RH, Vinegoni C, Weissleder R (2012). Neoplasia.

[15] Selvaraj R, Liu S, Hassink M, Huang CW, Yap LP, Park R, Fox JM, Li Z, Conti PS (2011). Bioorg. Med. Chem. Lett.

[16] Devaraj NK, Weissleder R, Hilderbrand SA (2008). Bioconjugate Chem.

[17] Nielsen O, Buchardt O (1991). Synthesis.

[18] Hara M, Wang X, Kawamura T, Bindokas VP, Dizon RF, Alcoser SY, Magnuson MA, Bell GI (2003). Am. J. Physiol. Endocrinol Metab.

[19] Degn KB, Brock B, Juhl CB, Djurhuus CB, Grubert J, Kim D, Han J, Taylor K, Fineman M, Schmitz O (2004). Diabetes.

[20] Orlando R, Floreani M, Padrini R, Palatini P (1998). Br. J. Clin. Pharmacol.

[21] Schmidt MM, Wittrup KD (2009). Mol. Cancer Ther.

[22] Runge S, Thogersen H, Madsen K, Lau J, Rudolph R (2008). J. Biol. Chem.

[23] Kagan L, Abraham AK, Harrold JM, Mager DE (2010). Pharm. Res.

[24] Thurber GM, Weissleder R (2011). PLoS One.

[25] Karver MR, Weissleder R, Hilderbrand SA (2011). Bioconjugate Chem.

[26] Karver MR, Weissleder R, Hilderbrand SA (2012). Angew. Chem.

[27] Li Z, Cai H, Hassink M, Blackman ML, Brown RC, Conti PS, Fox JM (2010). Chem. Commun.

[28] Chalkley HW, Cornfield J, Park H (1949). Science.

